# Antiobesity, Regulation of Lipid Metabolism, and Attenuation of Liver Oxidative Stress Effects of Hydroxy-*α*-sanshool Isolated from *Zanthoxylum bungeanum* on High-Fat Diet-Induced Hyperlipidemic Rats

**DOI:** 10.1155/2019/5852494

**Published:** 2019-08-27

**Authors:** Li Wang, Wenxiang Fan, Mengmeng Zhang, Qing Zhang, Lin Li, Jiaolong Wang, Lei Zhu, Daneng Wei, Wei Peng, Chunjie Wu

**Affiliations:** School of Pharmacy, Chengdu University of Traditional Chinese Medicine, Chengdu, China

## Abstract

*Zanthoxylum bungeanum* is a traditional Chinese medicine (TCM) used to relieve pain, dispel dampness, stop diarrhea, and prevent itching. The aim of this study was to investigate the antiobesity and hypolipidemic effects of hydroxy-*α*-sanshool (HAS) isolated from *Z. bungeanum* on hyperlipidemic rats. Wistar rats (*n* = 48) were randomly divided into six groups: (1) normal diet rats (ND), (2) high-fat diet- (HFD-) treated rats, (3) HFD+fenofibrate-treated rats (HFD+FNB), (4) HFD+low dose of HAS-treated rats (HFD+LD, 9 mg/kg), (5) HFD+middle dose of HAS-treated rats (HFD+MD, 18 mg/kg), and (6) HFD+high dose of HAS-treated rats (HFD+HD, 36 mg/kg). The body weight and food intake of the rats were recorded during the treatment period. After 4 weeks of HAS treatment, abdominal adipose tissues were observed and total cholesterol (T-CHO), triglycerides (TG), high-density lipoprotein (HDL) cholesterol (HDL-C), and low-density lipoprotein (LDL) cholesterol (LDL-C) of serum and liver tissues were determined. Furthermore, histochemical examinations using oil red O and hematoxylin-eosin staining (H&E) were carried out and levels of malondialdehyde (MDA) and glutathione (GSH) and activities of superoxide dismutase (SOD) in the liver were determined. After HFD feeding, the body weight gain and food efficiency ratio of HFD rats were significantly enhanced (*p* < 0.05*vs.* ND rats) and HAS treatment (18 and 36 mg/kg) significantly decreased the body weight gain and food efficiency ratio (*p* < 0.05*vs.* HFD rats). In addition, HAS treatment could decrease the abdominal adipose tissues and liver adipocytes. Furthermore, HAS treatment significantly decreased the T-CHO, TG, and LDL-C, whereas it increased HDL-C (*p* < 0.05*vs.* HFD rats) in serum and the liver. HAS treatment increased the GSH level and SOD activity in the liver (*p* < 0.05*vs.* HFD rats), whereas it decreased the levels of MDA (*p* < 0.05*vs.* HFD rats). mRNA analyses suggested that HAS treatment increases the expression of *Pparg* (proliferator-activated receptor *γ*) and *Apoe* (peroxisome apolipoprotein E). Immunohistochemistry and Western blotting indicated that HAS stimulation increased the levels of PPAR*γ* and APOE in the liver, as a stress response of the body defense system. These results revealed that HAS exerts antiobesity and hypolipidemic activities in HFD rats by reducing liver oxidative stress and thus could be considered as a potential candidate drug to cure or prevent obesity and hyperlipidemia.

## 1. Introduction

Cardiovascular diseases (CVDs), such as atherosclerosis (AS) and coronary heart disease (CHD), have become a global epidemic with high morbidity and mortality. Previous studies have shown that obesity and hyperlipidemia are positive risk factors for the initiation of atherosclerosis, which lead to a series of cardiovascular complications. Hyperlipidemia is a metabolic disorder disease that involves abnormally high levels of blood lipids and lipoproteins [1–3]. With the globalization of Western diet, a high-fat and high-cholesterol diet is believed to be one of the most important causes of the high incidence of obesity and hyperlipidemia worldwide [4]. Furthermore, the increased formation of free radicals and reactive oxygen species (ROS) participates in cardiac dysfunction, CVD progression, and cardiac apoptosis [3]. Oxidative stress occurs when the concentration of free radicals exceeds a critical level and the homeostasis of the body is disturbed [5]. Malondialdehyde (MDA), as an iconic product of oxidative stress, accumulates in the body and is toxic to cells, while superoxide dismutase (SOD) is an important part of the oxidation protection system that catalyzes the dismutation of the superoxide radical, thereby alleviating oxidative stress [5, 6]. The tripeptide glutathione (GSH) is also the most important cellular antioxidant [4]. Thus, attenuation of oxidative stress may be an effective preventive measure against hyperlipidemia. Current antihyperlipidemic medicines mainly include statins, fibrates, and bile acid chelators; however, they are inefficient in regulating lipid metabolism and attenuating oxidative stress. In addition, they also cause serious adverse effects such as rhabdomyolysis [3]. Therefore, the identification of a lipid-lowering drug with antioxidant potential from plants has attracted research interest. A recent study indicated that capsaicin extracted from the red chili pepper is beneficial for hyperlipidemia and atherosclerosis in guinea pigs fed a high-fat diet [7]. Furthermore, tetrahydrocurcumin intervention therapy has been proved to have therapeutic effects against obesity and hepatic steatosis [8].

**Figure 1:**
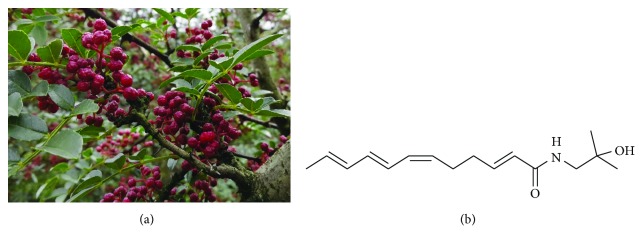
*Zanthoxylum bungeanum* (a) and hydroxy-*α*-sanshool (b).

*Zanthoxylum bungeanum* Maxim (*Z. bungeanum*) (Figure 1(a)), belonging to the family of Rutaceae, is used as a seasoning spice in China because of its unique aroma and taste [9, 10]. *Z. bungeanum* is also used as an effective traditional Chinese medicine to relieve pain, dispel dampness, stop diarrhea, and prevent itching [10, 11]. Previous phytochemical research reported that the main constituents of *Z. bungeanum* are unsaturated fatty acid amides of sanshool, such as hydroxy-*α*-sanshool, hydroxy-*β*-sanshool, and hydroxy-*γ*-sanshool [9, 11]. Pharmacological investigations revealed that *Z. bungeanum* possesses various pharmacological activities, in particular hypolipidemic and hypoglycemic effects [11, 12]. However, no active monomers corresponding to the hypolipidemic effect of *Z. bungeanum* have been reported. Consequently, as part of our continuing investigation into *Z. bungeanum*, we aimed to investigate the antiobesity and hypolipidemic effects of hydroxy-*α*-sanshool (HAS) (Figure 1(b)) isolated from *Z. bungeanum* on hyperlipidemic rats induced by high-fat diets. The present study would be beneficial for the development of HAS as a candidate drug to treat obesity and hyperlipidemia in the clinic.

## 2. Materials and Methods

### 2.1. Chemicals

The total cholesterol (T-CHO), triglycerides (TG), high-density lipoprotein (HDL) cholesterol (HDL-C), low-density lipoprotein (LDL) cholesterol (LDL-C), superoxide dismutase (SOD), malondialdehyde (MDA), tripeptide glutathione (GSH), and coomassie bright blue (CBB) kits were purchased from Nanjing Jiancheng Bioengineering Institute (Nanjing, China); rabbit anti-PPAR*γ* (proliferator-activated receptor *γ*) and rabbit anti-APOE (peroxisome apolipoprotein E) antibodies were products of Bioss Antibodies (Beijing, China); GAPDH antibodies, radioimmunoprecipitation assay (RIPA) lysate buffer, bicinchoninic acid (BCA) protein quantitative kit, and SDS-polyacrylamide gel electrophoresis (SDS-PAGE) preparation kit were purchased from Multi Sciences (Hangzhou, China); RNA TRIzol Reagent was purchased from Servicebio Company (Wuhan, China); the RevertAid First Strand cDNA Synthesis Kit was purchased from Thermo Fisher (MO, USA); and the all other chemicals used in this study were of analytical reagent grade.

### 2.2. Plant Material

The dried fruits of *Z. bungeanum* were collected from Qingxi town, Hanyuan County (Ya'an, China), and identified by Professor Chunjie Wu (School of Pharmacy, Chengdu University of Traditional Chinese Medicine, Chengdu, China). The voucher specimen of the fruit of *Z. bungeanum* was deposited at our laboratory (no. 20180317-1#).

### 2.3. Preparation of HAS

**Figure 2:**
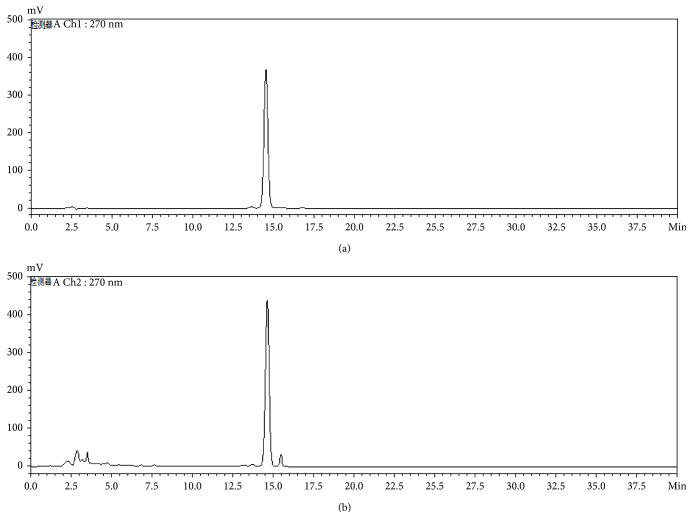
High-performance liquid chromatography (HPLC) analysis of the hydroxy-*α*-sanshool (HAS) extracted from the fruit of *Z. bungeanum*. (a) HPLC chromatogram of the standard; (b) HPLC chromatogram of the HAS extracted from the fruit of *Z. bungeanum*.

Fruits of *Z. bungeanum* were extracted twice with 20 volumes of 90% methanol (*v*/*v*) using a 1.5 h reflux extraction and subsequently concentrated under reduced pressure at 45°C. The concentrated extracts were then extracted by ethyl acetate three times and filtered, and then the solvent was vaporized to obtain the ethyl acetate fraction. The ethyl acetate fraction was then subjected to repeated column chromatography over silica gel (100–200 mesh) and eluted with petroleum ether-ethyl acetate (3 : 1-1 : 2) to yield four subfractions (sub-Fra. A-D). The sub-Fra. C was further subjected to preparative high-performance liquid chromatography (HPLC) with a CAPCELL PAK C18 MGII-F92539 (250 mm × 20 mm, 5 *μ*m; OSAKA SODA Co. Ltd., Osaka, Japan) column and eluted with acetonitrile in water (40 : 60) to yield the HAS monomer. Furthermore, HPLC analysis with an HAS standard was carried out to determine the authenticity and purity of the HAS. The Shimadzu LC-2010A system (Shimadzu Co., Japan) with a CAPCELL PAK C18 MGII-S5 column (4.6 mm × 250 mm, 5 *μ*m; OSAKA SODA Co. Ltd.) was used to perform the HPLC analysis, and acetonitrile (A)/water (B) = 40 : 60 was used as the mobile phase. The separation temperature was maintained at a constant 25°C for 40 min, with a flow rate of 1.0 mL/min and an injection volume of 10 *μ*L. Peaks were identified by comparing retention times and UV spectra with those of commercial standards and were quantified based on peak areas at 270 nm; the purity of the HAS was over 96.0% (Figure 2).

### 2.4. Toxicity Tests

A total of 60 Wistar rats were randomly divided into six groups (*n* = 10). Rats in groups 1–5 were orally administered 4.5, 9, 18, 36, and 72 mg/kg of HAS, respectively, and rats in the 6^th^ group received 20 mL/kg of normal saline. The mortality rates of the testing rats within a 72 h observation period were recorded. Furthermore, our results indicated that neither death nor any abnormal neurobehaviors could be observed with the doses ranging from 4.5 to 72 mg/kg during 72 h. Therefore, the 50% lethal dose (LD_50_) was not obtained and the safe doses (9, 18, and 36 mg/kg) were selected as administration dosages in our present study for the testing rats.

### 2.5. Animal Protocols

All animal experiments were conducted under protocols approved by the experimental animal ethics committee of Chengdu University of Traditional Chinese Medicine. Wistar rats (220 ± 20 g) were purchased from the Chengdu *Dashuo* Experimental Animals Co. Ltd. (Chengdu, China) and housed in a facility maintained at a constant temperature (22 ± 1°C) and humidity (55 ± 10%) with an alternating 12 hour light/dark cycle. All rats were provided with *ad libitum* access to water and standard rodent chow for 1 week before performing the experiments. In total, 48 Wistar rats were randomly divided into six groups (*n* = 8). Besides the normal diet (ND) group, rats in the other five groups were fed with high-fat diet (HFD) feeds (consisting of basic feed (73%), cholesterol (1.5%), pig fat (10%), egg yolk powder (5%), sucrose (10%), and bile salt (0.5%)).

**Figure 3:**
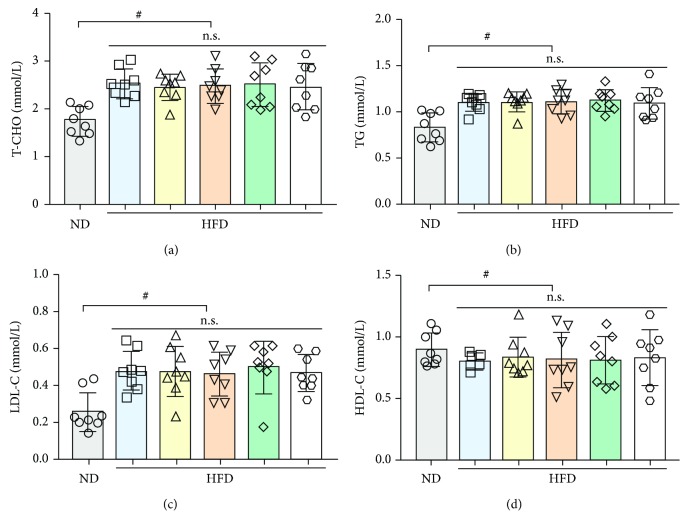
Total cholesterol (T-CHO) (a), total glycerides (TG) (b), low-density lipoprotein cholesterol (LDL-C) (c), and high-density lipoprotein chol esterol (HDL-C) (d) in rat serum consuming a normal diet (ND) and a high-fat diet (HFD) for two weeks. Values are expressed as the mean ± SD (*n* = 8), ^#^*p* < 0.05*vs*. ND; n.s.: nonsignificant.

After two weeks of HFD feeding, to evaluate whether the hyperlipidemic rats were prepared successfully, the total cholesterol (T-CHO), triglycerides (TG), high-density lipoprotein (HDL) cholesterol (HDL-C), and low-density lipoprotein (LDL) cholesterol (LDL-C) in the rats' serum were determined. Briefly, blood samples were collected from rat tail veins and centrifuged at 4°C (2500 rpm, 15 min) to provide the serum samples for the biochemical assays, which were performed using commercial testing kits according to the manufacturer's instructions. As shown in Figure 3, the levels of T-CHO (*p* < 0.05), TG (*p* < 0.05), and LDL-C (*p* < 0.05) in serum increased significantly, whereas the HDL-C levels decreased significantly (*p* < 0.05), compared with those in the ND rats. These results suggested that the hyperlipidemic rats were prepared successfully and could be used for further experiments.

**Figure 4:**
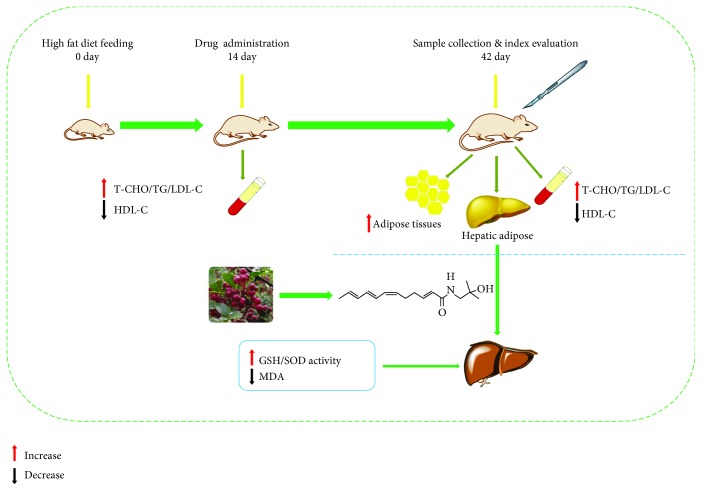
Schematic diagram of the experiment.

The HFD-treated rats were adjusted and subdivided into five groups (*n* = 8 in each group) according to the body weight of the rats and the biochemical values in serum. Subsequently, the HAS and fenofibrate (FNB) were dissolved in 0.5% carboxymethyl cellulose (CMC) -Na solution and all the tested drugs were administered orally; rats were treated with FNB (HFD+FNB, 18 mg/kg), a low dose of HAS (HFD+LD, 9 mg/kg), a middle dose of HAS (HFD+MD, 18 mg/kg), and a high dose of HAS (HFD+HD, 36 mg/kg) for 4 weeks. Rats in the ND and HFD groups were treated with an equivalent volume of 0.5% CMC-Na solution. The rats' body weights were measured every week, and food intake was recorded every day during the treatment period. After four weeks of treatment, the rats were anesthetized with 2% (*v*/*v*) sodium pentobarbital (3 mL/kg) and then blood samples were collected from the abdominal aorta. The rats' abdomens were opened for observation and to isolate the total abdominal fat; all rats were then sacrificed by cervical dislocation after blood sampling. The liver tissues were collected rapidly and cut into three pieces; one piece was sectioned into small pieces (1 cm × 1 cm) and fixed with 4% paraformaldehyde, one piece was frozen in liquid nitrogen, and the last piece was stored at −20°C for further biochemical analyses. In addition, blood samples were centrifuged at 2500 rpm under 4°C for 15 min to acquire serum samples and subsequently stored at −70°C for subsequent biochemical analyses. The experimental scheme is shown in Figure 4.

### 2.6. Biochemical Analysis in Serum and the Liver

The contents of HDL-C and LDL-C in serum and T-CHO and TG in the serum and liver tissues were measured using commercially available kits according to the manufacturer's instructions (Nanjing Jiancheng Bioengineering Institute), and a microplate reader was used to read the optical density (OD) values (BIO-BRI Technology Co. Ltd., Chengdu, China).

### 2.7. Oxidative Stress Marker Analysis in Liver Tissues

The levels of MDA and GSH and the activities of (SOD) in hepatic tissues were determined using commercially available colorimetric kits (Nanjing Jiancheng Bioengineering Institute) according to the manufacturer′s instructions. Meanwhile, a CBB kit (Nanjing Jiancheng Bioengineering Institute) was used out to measure the total content of protein in 10% liver homogenate.

### 2.8. Oil Red O Staining

The liver tissue sections were fixed with formaldehyde for 10 min, washed with distilled water, and soaked with 60% isopropyl alcohol. The fixed sections were then stained with oil red O solution for 10 min and subjected to hematoxylin staining. The lipid droplets were visualized using a digital trinocular camera microscope (BA400 Digital, McAudi).

### 2.9. Hematoxylin and Eosin Staining

The liver tissue sections were dewaxed, stained with hematoxylin for 10–20 min, and rinsed with tap water for 1–3 min. The sections were then incubated with hydrochloric acid alcohol for 5–10 s, rinsed with tap water, and placed in a weakly alkaline aqueous solution until a blue color appeared. The sections were then placed in 85% alcohol for 3–5 min, stained with eosin for 3–5 min, and then washed for 3–5 s. Serial dilutions of ethyl alcohol were used for dehydration, and the sections were cleared in xylene and sealed using neutral glycerin. Images were acquired using a digital trinocular camera microscope (BA400 Digital, McAudi).

### 2.10. Immunohistochemistry (IHC)

The liver tissue sections were dewaxed and incubated in 3% methanol-hydrogen peroxide (3 : 97, *v*/*v*) for 10 min and then washed with phosphate-buffered saline (PBS) three times for 5 min each time. The sections were then immersed in citrate buffer (pH 6.0), microwaved until boiling, repeated once, and washed with PBS two times for 5 min each time. After blocking with goat serum at room temperature for 20 min, the tissue sections were incubated with rabbit anti-PPAR*γ* (proliferator-activated receptor *γ*) (1 : 50; cat. no. bs-0167R, Bioss) and rabbit anti-APOE (peroxisome apolipoprotein E) antibodies (1 : 100; cat. no. bs-3614R, Bioss) at 4°C overnight, followed by incubation with a secondary antibody (cat. no. SP-9001, Beijing Zhongshan Jinqiao Biological Co. Ltd., Beijing, China) at 37°C for 30 min. Specific labeling was visualized using a 3,3′-diaminobenzidine (DAB) kit and appeared yellow or brownish yellow, while cell nuclei were counterstained with hematoxylin (blue staining). Images were captured under a digital trinocular camera microscope (BA400 Digital, McAudi), and in each slide, three random images of the liver were obtained to determine the intensity of staining. IHC staining was expressed as the relative positive expression, and the mean density determined by the ImageJ software (version: 1.51, National Institutes of Health, MD, USA) was used to compare the potential differences between groups.

### 2.11. Quantitative Real-Time PCR

The levels of *Apoe* mRNA *and Pparg* mRNA expression in the liver were measured by the quantitative real-time PCR. The liver tissue was homogenized, and total RNA was isolated using the TRIzol Reagent (G3013, Servicebio) according to the manufacturer's instructions. To synthesize cDNA, 2 *μ*g of total RNA was mixed with 1 *μ*L of oligo (dT) 18 and diethyl pyrocarbonate-treated water to a final volume of 12 *μ*L. The mixture was incubated at 65°C for 5 min and cooled on the ice rapidly. Then 4 *μ*L of 5x Reaction Buffer, 2 *μ*L of 10 mM dNTP Mix, 1 *μ*L of RiboLock RNAase Inhibitor (20 U/*μ*L), and 1 *μ*L of RevertAid M-MuLV Reverse Transcriptase (200 U/*μ*L) were added successively. The mixture was incubated at 42°C for 60 min, and the reaction was stopped by heat inactivation at 70°C for 5 min. Quantitative real-time PCR was performed using a fluorescent quantitative PCR (Thermo Fisher Scientific). Reactions were carried out according to the following protocol: heating to 95°C for 10 min, followed by 40 cycles of 95°C for 15 s and 60°C for 60s. *Gapdh* (Thermo Fisher) was used as the internal control gene to normalize the fold change in gene expression in the different groups. Primers for the target genes were designed by Sangon Bioengineering (Shanghai) Co. Ltd. (Shanghai, China) using Primer 3 software based on sequences retrieved from the GenBank database (Table 1).

### 2.12. Western Blot Analysis

Western blotting was used to measure the liver level of PPAR*γ* and APOE. About 5 mg of liver tissue was placed in radioimmunoprecipitation assay (RIPA) strong lysate buffer (RIPA+1% PMSF) and pulverized. The total protein was extracted using a BCA protein quantitative kit (cat. no. 70-pq0011, Multi Science) according to the manufacturer's protocol. Protein samples were separated using 10% SDS-PAGE and then transferred to polyvinylidene fluoride (PVDF) membranes. Then the PVDF membranes were blocked by 5% skimmed milk. After that, the PVDF membranes were incubated by the primary antibodies of PPAR*γ* (dilution: 1 : 1000), APOE (dilution: 1 : 1000), and GAPDH (dilution: 1 : 1000) overnight at 4°C and subsequently incubated by horse radish peroxidase-conjugated secondary antibody for 1 h. The target protein bands were visualized by chemiluminescence detection with BeyoECL Star kits (Beyotime Biotech. Co., Haimen, China), and the protein band densities were determined by the ImageJ software (version: 1.51, National Institutes of Health); additionally, the protein expression levels of PPAR*γ* and APOE were normalized to those of GAPDH.

### 2.13. Statistical Analysis

All results were expressed as the mean±the standard deviation (SD). The differences among the experimental groups were evaluated using one-way analysis of variance (ANOVA), and significant differences among the means were determined using Duncan's multiple-range tests. *p* values less than 0.05 were considered statistically significant.

## 3. Results

### 3.1. Effects of HAS on Body Weight, Food Intake, Weight Gain, and the Food Efficiency Ratio

**Figure 5:**
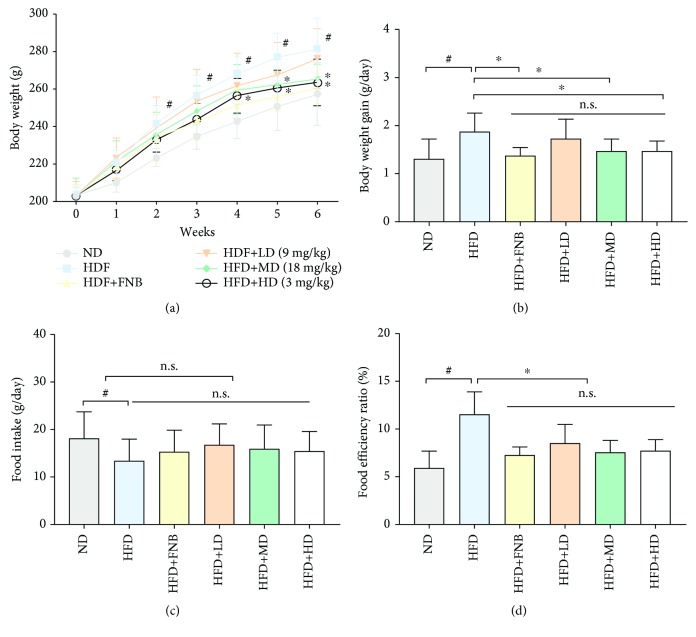
Effect of hydroxy-*α*-sanshool (HAS) on body weight (a), body weight gain (b), food intake (c), and food efficiency ratio (d) in rats consuming a high-fat diet. We measured body weight every week for 6 weeks. The food efficiency ratio is the daily weight gain divided by the daily food intake. Values are expressed as the mean ± SD (*n* = 8); ND: normal diet-treated rats; HFD: high-fat diet-treated rats; HFD+FNB: HFD supplemented with fenofibrate-treated rats; HFD+LD: rats treated with HFD supplemented with a low dose of HAS; HFD+MD: rats treated with HFD supplemented with a middle dose of HAS; HFD+HD: rats treated with HFD supplemented with a high dose of HAS; ^#^*p* < 0.05*vs.* ND; ^∗^*p* < 0.05*vs.* HFD; n.s.: nonsignificant.

After 2 weeks of HFD feeding, the body weights of the HFD rats were significantly higher than those of the ND rats (*p* < 0.05) (Figure 5(a)) and were similar to the positive drug-treated rats (fenofibrate, 18 mg/kg). HAS could decrease the body weight of HFD rats at doses of 18 and 36 mg/kg (*p* < 0.05) (Figure 5(a)). Furthermore, the results showed that the body weight gain (BWG) and food efficiency ratio (FER) were sharply increased by HFD feeding at the end of the experimental period (both *p* < 0.05), compared with those of ND rats (Figures 5(b) and 5(d)). Interestingly, both FNB (18 mg/kg) and HAS (18 and 36 mg/kg) significantly reduced the BWG (Figure 5(b)) and FER (Figure 5(d)) of HFD-fed rats (*p* < 0.05). The food intake of the HFD rats was lower than that of the ND rats; however, HAS (18 and 36 mg/kg) treatment had no obvious effect on the food intake of the HFD rats (Figure 5(d)).

### 3.2. Effect of HAS on Abdominal Adipose Tissue

**Figure 6:**
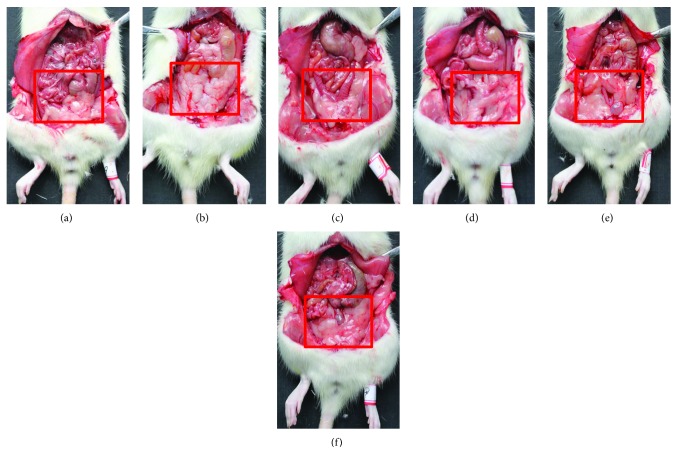
Effect of HAS on the abdominal adipose tissue in HFD rats. (a–f) represent the amount of abdominal adipose tissues of rats in the ND, HFD, HFD+FNB, HFD+LD, HFD+MD, and HFD+HD groups, respectively. ND: normal diet-treated rats; HFD: high-fat diet-treated rats; HFD+FNB: HFD supplemented with fenofibrate-treated rats; HFD+LD: rats treated with HFD supplemented with a low dose of HAS; HFD+MD: rats treated with HFD supplemented with a middle dose of HAS; HFD+HD: rats treated with HFD supplemented with a high dose of HAS.

As can be seen from Figure 6 and Table 2, after HFD feeding for 6 weeks, the HFD rats had much more white abdominal adipose tissue than the ND rats (Figures 6(a) and 6(b); *p* < 0.05). For the FNB-treated rats (18 mg/kg), the amount of white abdominal adipose tissue was reduced compared with that in the HFD rats (*p* < 0.05). Similar to FNB (Figure 6(c)), HAS at doses of 9, 18, and 36 mg/kg decreased the amount of white abdominal adipose tissues of HFD rats (Figures 6(d)–6(f); all *p* < 0.05).

### 3.3. Effect of HAS on Lipid Levels in Serum and Liver Tissues

From the results shown in Table 3, we observed that the levels of T-CHO and TG in the serum and liver tissues increased significantly (all *p* < 0.05) compared with those in the ND rats. Importantly, the LDL-C level in serum increased (*p* < 0.05) sharply after HFD feeding, whereas the HDL-C levels markedly decreased (*p* < 0.05). Interestingly, treatment with FNB (18 mg/kg, *p* < 0.05) or HAS (18 and 36 mg/kg; both *p* < 0.05) could significantly decrease the T-CHO and TG levels in both serum and the liver, compared with those in the rats in the HFD group. Furthermore, the results also indicated that FNB (18 mg/kg, *p* < 0.05) and HAS (9, 18, and 36 mg/kg; all *p* < 0.05) could ameliorate the increased LDL-C in the serum of HDF-fed rats, whereas FNB (18 mg/kg, *p* < 0.05) and HAS (18 mg/kg, *p* < 0.05) increased the serum level of HDL-C.

### 3.4. Results of the Histopathological Examinations

**Figure 7:**
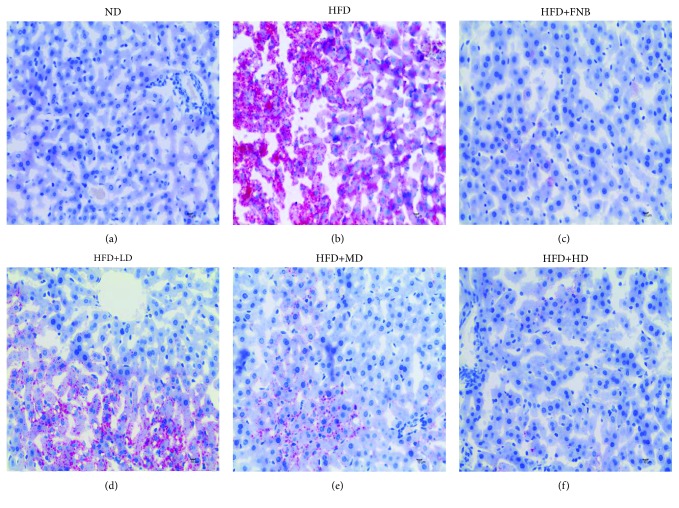
Histopathological examinations of liver tissues with oil red O staining. (a–f) represent oil red O-stained liver sections of rats in the ND, HFD, HFD+FNB, HFD+LD, HFD+MD, and HFD+HD groups, respectively. ND: normal diet-treated rats; HFD: high-fat diet-treated rats; HFD+FNB: HFD supplemented with fenofibrate-treated rats; HFD+LD: rats treated with HFD supplemented with a low dose of HAS; HFD+MD: rats treated with HFD supplemented with a middle dose of HAS; HFD+HD: rats treated with HFD supplemented with a high dose of HAS.

To determine the improving effects of HAS on the histopathological changes of liver tissues of hyperlipidaemic rats induced by HFD feeding, oil red O and H&E staining experiments were carried out. As shown in Figure 7, preadipocytes were stained with the fat-specific oil red O several days after the induction of hepatic adipocyte differentiation. The results revealed that HFD feeding markedly increase adipocyte differentiation compared with that in the ND rats (Figures 7(a) and 7(b)). In addition, the results indicated that FNB (18 mg/kg, Figure 7(c)) and HAS (9, 18, and 36 mg/kg, Figures 7(d)–7(f)) significantly decreased the adipocyte ratio in the liver tissues of HFD rats.

**Figure 8:**
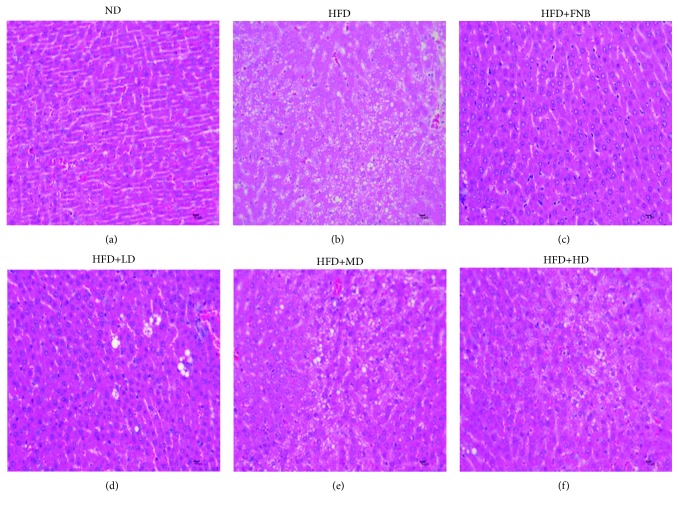
Histopathological examinations of liver tissues using H&E staining. (a–f) represent H&E-stained liver sections of rats in the ND, HFD, HFD+FNB, HFD+LD, HFD+MD, and HFD+HD groups, respectively. ND: normal diet-treated rats; HFD: high-fat diet-treated rats; HFD+FNB: HFD supplemented with fenofibrate-treated rats; HFD+LD: rats treated with HFD supplemented with a low dose of HAS; HFD+MD: rats treated with HFD supplemented with a middle dose of HAS; HFD+HD: rats treated with HFD supplemented with a high dose of HAS.

The results of the histopathological examinations of liver tissues using H&E staining are shown in Figure 8. In the ND group, normal liver architecture and hepatic lobules were observed (Figure 8(a)), while liver necrosis, interstitial edema, vacuolization of hepatocytes, and severe accumulation of lipid droplet in hepatocytes were observed in the HFD-fed rats (Figure 8(b)). However, FNB treatment (18 mg/kg) alleviated the pathological changes in liver tissues; especially, no obvious lipid droplets in hepatocyte were observed (Figure 8(c)). Similar to the effects of FNB, HAS (9, 18, and 36 mg/kg) also ameliorated lipid droplet accumulation in hepatocytes of HFD-fed rats (Figures 8(d)–8(f)).

### 3.5. Effect of HAS on Oxidative Stress Markers in Liver Tissues

The effects of HAS on MDA, GSH, and SOD in liver tissues are shown in Table 4. The results showed that HFD feeding decreased the levels of SOD and GSH and increased that of MDA in liver tissues (all *p* < 0.05), compared with those in the ND rats. Interestingly, the results suggested that the HAS and FNB could increase the levels of GSH and SOD and decrease the levels of MDA (all *p* < 0.05).

### 3.6. Effect of HAS on the Levels of PPAR*γ* and APOE in Liver Tissues through IHC

**Figure 9:**
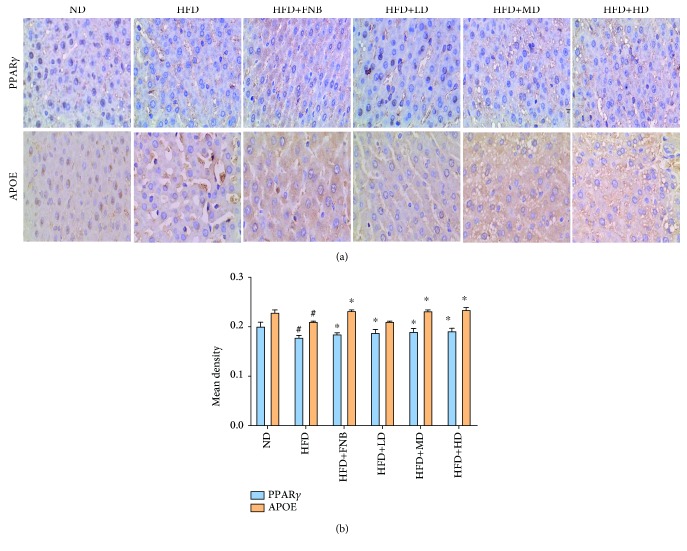
Effect of HAS on the expression of PPAR*γ* and APOE in liver tissues using IHC. ND: normal diet-treated rats; HFD: high-fat diet-treated rats; HFD+FNB: HFD supplemented with fenofibrate-treated rats; HFD+LD: rats treated with HFD supplemented with a low dose of HAS; HFD+MD: rats treated with HFD supplemented with a middle dose of HAS; HFD+HD: rats treated with HFD supplemented with a high dose of HAS; ^#^*p* < 0.05 vs. ND; ^∗^*p* < 0.05 vs. HFD; n.s.: nonsignificant.

As shown in Figure 9(a), the PPAR*γ* and APOE protein levels were enriched in the nuclear fraction and in the cytoplasmic fraction in liver cells using different doses of HFD+FNB and HFD+HAS (9 mg/kg, 18 mg/kg, and 36 mg/kg) compared with those in the HFD groups (Figure 9(b), all *p* < 0.05). The increased levels of PPAR*γ* suggested that the HAS might activate the PPAR*γ* pathway. Furthermore, the results also indicated that HAS and FNB could increase the expression levels of APOE compared with those in the HFD rats (Figure 9(b)).

### 3.7. Effect of HAS on the Transcription of Genes Involved in Lipid Metabolism in Liver Tissues

**Figure 10:**
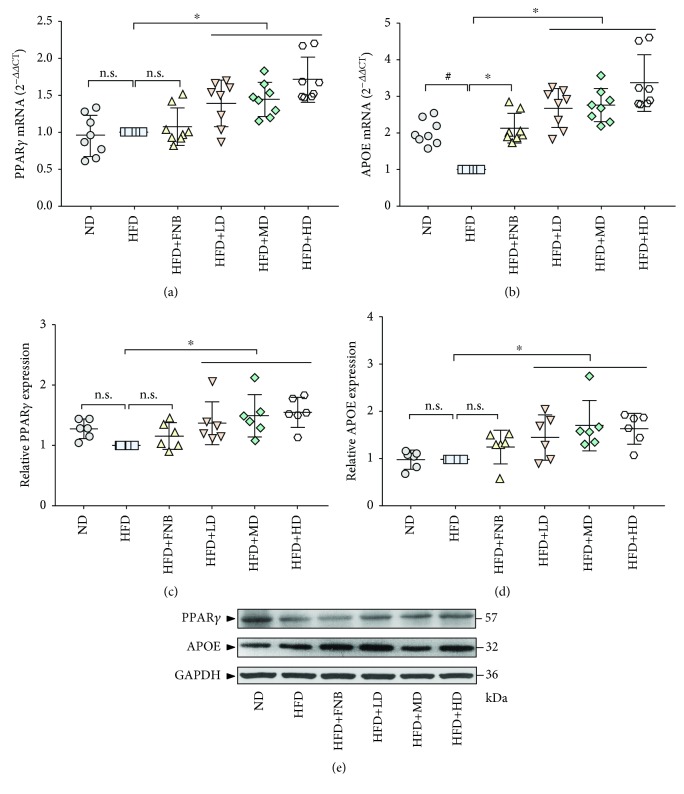
Effect of HAS on the transcription of genes involved in lipid metabolism in liver tissues (a, b); values are expressed as mean ± SD (*n* = 9). Effect of HAS on the levels of PPAR*γ* and APOE in liver tissue using Western blotting (c–e). Values are expressed as mean ± SD (*n* = 6). ND: normal diet-treated rats; HFD: high-fat diet-treated rats; HFD+FNB: HFD supplemented with fenofibrate-treated rats; HFD+LD: rats treated with HFD supplemented with a low dose of HAS; HFD+MD: rats treated with HFD supplemented with a middle dose of HAS; HFD+HD: rats treated with HFD supplemented with a high dose of HAS; ^#^*p* < 0.05*vs*. ND; ^∗^*p* < 0.05*vs*. HFD; n.s.: nonsignificant.

The mRNA expression of adipocyte markers, such as *Apoe*, was decreased in the liver tissues of HFD rats compared with those in the ND rats (*p* < 0.05, Figure 10(a)), while the changes in *Pparg* expression were nonsignificant (Figures 10(a) and 10(b)). Furthermore, the results indicated that HAS (9, 18, and 36 mg/kg; all *p* < 0.05) could increase the expression of *Pparg* and *Apoe* mRNA compared with that in the HFD rats. FNB could also increase the expression of the *Apoe* mRNA compared with that in the HFD rats (Figure 10(b), *p* < 0.05), while the change in *Pparg* expression was nonsignificant (Figure 10(a)).

### 3.8. Effect of HAS on PPAR*γ* and APOE Levels in Liver Tissue

As shown in Figure 10, the levels of PPAR*γ* in the HFD group were significantly lower than those in the ND group under high-fat conditions (Figures 10(c) and 10(e)). The levels of all detected proteins were higher in the HFD+HAS groups (9, 18, and 36 mg/kg) compared with those in the HFD group (Figures 10(c)–10(e); all *p* < 0.05), while the changes resulting from FNB treatment were nonsignificant.

## 4. Discussion

Traditional Chinese medicines (TCMs) have been used comprehensively to treat various diseases for thousands of years in Chinese folk medicine [13–15]. In addition, plants are the predominant sources of food for humans and it is reported that over half of the available drugs are derived from natural products derived from plants [16, 17]. Consequently, searching for candidate agents with reliable antiobesity and hypolipidemic activities from plants is a feasible approach. Importantly, in the present study, we reported that hydroxy-*α*-sanshool (HAS) isolated from *Z. bungeanum* has potential antiobesity and hypolipidemic activities and attenuates liver oxidative stress effects.

Obesity is generally recognized as a chronic metabolic disorder and is characterized by excess body fat and weight induced by imbalance of energy consumption and intake [18]. Currently, obesity is believed to be closely related to high-fat diets, lack of exercise, and genetics [13, 19]. In the present study, we prepared fatty and hyperlipidemic rats fed with high-fat diets, which had similar pathological characteristics to patients with obesity and hyperlipidemia. The results showed that compared with the model rats, treatment with HAS not only significantly decreased body weight gain but also reduced the underbelly visceral fat of the HFD-fed rats. In addition, fatty liver is a common complication of obesity and hyperlipidemia [20]. Based on the H&E and oil red staining histopathological examinations, HAS administration could markedly ameliorate the degree of fatty liver of the HFD-fed rats. This evidence demonstrated that HAS possesses potential antiobesity and hypolipidemic activities in HFD-fed rats.

Obesity induces lipid metabolism disorders, in particular the metabolism of TC, TG, LDL-C, and HDL-C. Clinically, patients with obesity and hyperlipidemia commonly present with increases in TC, TG, and LDL-C and decreased HDL-C [21, 22]. Interestingly, the results of the present study demonstrated that HAS administration could decrease the contents of TC, TG, and LDL-C and increase the HDL-C contents in HFD rats.

Moreover, increasing evidence indicates that oxidative stress is closely correlated with the development of obesity, fatty liver, hyperlipidemia, and arteriosclerosis. In addition, inhibition of oxidative stress might be beneficial to control obesity and hyperlipidemia and protecting liver tissue [23, 24]. MDA is one of the major end products of lipid peroxidation (LPO) and is commonly used as an indicator of liver tissue damage. In contrast, the antioxidant defense system in the body plays a vital role in inhibiting oxidative stress. GSH and SOD are crucial antioxidative enzymes [25] in liver tissues. Our study demonstrated that HAS administration could increase the levels of GSH and SOD and decrease the level of MDA in liver tissues. These results revealed that HAS could decrease the oxidative stress level in liver tissues.

Peroxisome proliferator-activated receptors (PPARs) play an important regulatory role in regulating the homeostasis of adipose tissue by regulating the balance between anabolic and oxidative processes [26]. PPARs are mainly expressed in white and brown adipose tissue, which plays an important anabolic role in promoting fat storage, fat formation, and thermogenesis [27, 28]. Furthermore, APOE, a protective protein for cardiovascular diseases, can interact with PPAR*γ* to decrease cardiovascular disease risk [29]. Interestingly, the results of the mRNA analyses suggested that the HAS treatment increases the expression of *Pparg* and *Apoe*. The IHC and Western blotting results also indicated that HAS treatment could increase the levels of PPAR*γ* and APOE in the nuclei, as a stress response of the body's defense system. These results indicated that HAS might regulate lipid metabolism and reduce oxidative stress in the high-fat state by upregulating *Pparg* expression. Meanwhile, HAS also increased the expression of *Apoe* and promoted the metabolism and transformation of lipoproteins.

## 5. Conclusion

In summary, hydroxy-*α*-sanshool (HAS) could play a significant role in improving the lipid profile and may act as a protective agent against atherosclerosis by regulating of PPAR*γ* and APOE to reduce lipid peroxidation. Therefore, HAS could be considered as a potential candidate drug to cure or prevent hyperlipidemia.

## Figures and Tables

**Table 1:** Sequences of primers used in qRT-PCR.

Genes		GenBank number	Fragment length (bp)
*Gapdh*			
F:	CTGGAGAAACCTGCCAAGTATG	NM_017008.4	138
R:	GGTGGAAGAATGGGAGTTGCT		
*Apoe*			
F:	TGACGGTACTGATGGAGGACACT	NM_001270681.1	201
R:	CCAGCATGGTGTTTACCTCGTT		
*Pparg*			
F:	CCCTTTACCACGGTTGATTTC	NM_001145366.1	321
R:	CTTCAATCGGATGGTTCTTCG		

F: forward; R: reverse.

**Table 2:** Effect of HAS on abdominal adipose tissue.

Groups	Abdominal adipose tissue (g)	Abdominal adipose tissue/final body weight (%)
ND	6.06 ± 0.80	2.35 ± 0.28
HFD	9.53 ± 0.90^#^	3.39 ± 0.31^#^
HFD+FNB	6.69 ± 0.58^∗^	2.57 ± 0.20^∗^
HFD+LD	7.96 ± 0.71^∗^	2.88 ± 0.22^∗^
HFD+MD	6.84 ± 0.53^∗^	2.58 ± 0.26^∗^
HFD+HD	6.55 ± 0.39^∗^	2.49 ± 0.21^∗^

Values are expressed as mean ± SD (*n* = 8); ^#^*p* < 0.05*vs*. the ND group and ^∗^*p* < 0.05*vs*. the HFD group. HAS: hydroxy-*α*-sanshool; ND: normal diet; HFD: high-fat diet; FNB: fenofibrate; LD: low dose of HAS; MD: medium dose of HAS; HD: high dose of HAS.

**Table 3:** Effect of HAS on lipid levels in serum and liver tissues of HFD rats.

Groups	Serum				Liver	
	Total cholesterol (mmol/L)	Triglyceride (mmol/L)	LDL cholesterol (mmol/L)	HDL cholesterol (mmol/L)	Total cholesterol (mmol/gprot)	Triglyceride (mmol/gprot)
ND	1.76 ± 0.28	0.88 ± 0.07	0.25 ± 0.07	1.10 ± 0.16	0.15 ± 0.01	0.22 ± 0.02
HFD	2.80 ± 0.52^#^	1.84 ± 0.18^#^	0.98 ± 0.16^#^	0.93 ± 0.11^#^	0.69 ± 0.16^#^	0.43 ± 0.07^#^
HFD+FNB	1.82 ± 0.56^∗^	1.03 ± 0.21^∗^	0.25 ± 0.07^∗^	1.12 ± 0.21^∗^	0.18 ± 0.03^∗^	0.26 ± 0.07^∗^
HFD+LD	2.39 ± 0.45	1.16 ± 0.13^∗^	0.66 ± 0.09^∗^	1.02 ± 0.19	0.45 ± 0.14^∗^	0.26 ± 0.05^∗^
HFD+MD	1.83 ± 0.29^∗^	1.13 ± 0.25^∗^	0.44 ± 0.14^∗^	1.08 ± 0.17^∗^	0.38 ± 0.11^∗^	0.23 ± 0.07^∗^
HFD+HD	1.98 ± 0.34^∗^	0.87 ± 0.15^∗^	0.29 ± 0.10^∗^	1.04 ± 0.11	0.26 ± 0.09^∗^	0.21 ± 0.02^∗^

Values are expressed as mean ± SD (*n* = 8); ^#^*p* < 0.05*vs*. the ND group and ^∗^*p* < 0.05*vs*. the HFD group. HAS: hydroxy-*α*-sanshool; ND: normal diet; HFD: high-fat diet; FNB: fenofibrate; LD: low dose of HAS; MD: medium dose of HAS; HD: high dose of HAS; LDL: low-density lipoprotein; HDL: low-density lipoprotein.

**Table 4:** Effect of HAS on MDA, GSH, and SOD in liver tissues.

Groups	SOD (U/mgprot)	MDA (*μ*mol/gprot)	GSH (*μ*mol/gprot)
ND	204.74 ± 15.52	3.69 ± 0.43	35.274 ± 2.26
HFD	147.83 ± 32.77^#^	5.84 ± 1.16^#^	26.583 ± 3.43^#^
HFD+FNB	214.40 ± 23.05^∗^	3.68 ± 0.79^∗^	36.120 ± 5.72^∗^
HFD+LD	152.01 ± 16.73^∗^	3.88 ± 093^∗^	29.371 ± 7.59
HFD+MD	207.60 ± 28.29^∗^	3.82 ± 081^∗^	30.210 ± 7.27
HFD+HD	216.32 ± 39.64^∗^	3.37 ± 061^∗^	34.722 ± 5.53^∗^

Values are expressed as mean ± SD (*n* = 8); ^#^*p* < 0.05*vs*. the ND group and ^∗^*p* < 0.05*vs*. the HFD group. HAS: hydroxy-*α*-sanshool; ND: normal diet; HFD: high-fat diet; FNB: fenofibrate; LD: low dose of HAS; MD: medium dose of HAS; HD: high dose of HAS; SOD: superoxide dismutase; MDA: malondialdehyde; GSH: glutathione.

## Data Availability

The data used to support the findings in this paper are available from the corresponding author upon request.
